# Human Thyroid Cancer-1 (TC-1) is a vertebrate specific oncogenic protein that protects against copper and pro-apoptotic genes in yeast

**DOI:** 10.15698/mic2015.07.213

**Published:** 2015-07-06

**Authors:** Natalie K. Jones, Nagla T. Arab, Rawan Eid, Nada Gharib, Sara Sheibani, Hojatollah Vali, Chamel Khoury, Alistair Murray, Eric Boucher, Craig A. Mandato, Paul G. Young, Michael T. Greenwood

**Affiliations:** 1 Department of Chemistry and Chemical Engineering, Royal Military College of Canada, Kingston, Ontario, Canada.; 2 Department of Anatomy and Cell Biology, McGill University, Montreal, Quebec, Canada.; 3 Department of Biology, Queen’s University, Kingston, Ontario, Canada.; 4 Present address: Department of Experimental Medicine, McGill University, Montreal, Quebec, Canada.; 5 Present address: Department of Biomedical Sciences, Queen’s University, Kingston, Ontario, Canada.; 6 Present address: Defence Research and Development Canada, Alberta, Canada.

**Keywords:** TC-1, apoptosis, cell death, anti-apoptotic, cell survival, cancer, yeast

## Abstract

The human Thyroid Cancer-1 (hTC-1) protein, also known as C8orf4 was initially identified as a gene that was up-regulated in human thyroid cancer. Here we show that hTC-1 is a peptide that prevents the effects of over-expressing Bax in yeast. Analysis of the 106 residues of hTC-1 in available protein databases revealed direct orthologues in jawed-vertebrates, including mammals, frogs, fish and sharks. No TC-1 orthologue was detected in lower organisms, including yeast. Here we show that TC-1 is a general pro-survival peptide since it prevents the growth- and cell death-inducing effects of copper in yeast. Human TC-1 also prevented the deleterious effects that occur due to the over-expression of a number of key pro-apoptotic peptides, including *YCA1*, *YBH3*, *NUC1*, and *AIF1*. Even though the protective effects were more pronounced with the over-expression of *YBH3* and *YCA1*, hTC-1 could still protect yeast mutants lacking *YBH3* and *YCA1 *from the effects of copper sulfate. This suggests that the protective effects of TC-1 are not limited to specific pathways or processes. Taken together, our results indicate that hTC-1 is a pro-survival protein that retains its function when heterologously expressed in yeast. Thus yeast is a useful model to characterize the potential roles in cell death and survival of cancer related genes.

## INTRODUCTION

Programmed cell death (PCD) is a biological process that is activated in response to a variety of stresses 1,2. Stresses can serve to activate both pro- and anti-apoptotic processes in order to destroy the cell or to prevent the cell from undergoing a pre-mature death 3. The most commonly studied form of genetically encoded cell death is controlled by the Bcl-2 family of proteins 4,5. Bax is a pro-death Bcl-2 family member that gets activated by a variety of stresses in order to initiate cell death processes. In apoptotic cell death, Bax leads to mitochondrial damage, increases in reactive oxygen species (ROS), release of apoptogenic mitochondrial factors including cytochrome *c*, AIF and EndoG that lead to controlled cell breakdown in part by the activation of caspases 6. The effects of activated Bax can be offset by the pro-survival Bcl-2 protein. In addition to inhibiting Bax, overexpressed Bcl-2 can prevent multiple forms of cell death including cell death that occurs in the absence of activated Bax 7. The ability to prevent multiple forms of cell death is a commonly observed for many pro-survival proteins 3.

Programmed cell death can occur via at least three different processes including apoptosis (type I), necrosis or necroptosis (type II) and autophagy (type III) 2,6. The complexity of PCD is further enhanced by the fact that there are multiple sub-forms of all PCD sub-types as well as by the existence of extensive cross-talk between the different forms 1,2,3. Many human diseases, such as ischemic heart and neurological diseases, are associated with abnormal signaling within the cell, which gives rise to altered levels of cell death. This has led to the development of therapeutic strategies aimed at increasing or decreasing the function of apoptotic regulators 4. Blocking specific apoptotic effectors like caspases have been shown to have limited success in blocking cell death in *in vivo* models 3,8. This is likely due to the activation of alternative cell death pathways or to the inability to interfere with cell death once the process has been initiated. Initially, apoptosis was thought to be the predominant form of cell death in many diseases, but it is now clear that other forms of cell death including necroptosis play important roles in the loss of cells 9,10,11. In contrast, other diseases such as cancer are associated with decreases in cell death responses 12,13,14. Increases in the protective effects of autophagy as well as increases in the expression levels of pro-survival proteins, such as Bcl-2, can promote changes in the apoptotic and PCD responses 12,15. Thus, instead of developing chemicals targeting specific apoptotic regulators, the development of strategies based on the knockdown or the overexpression of apoptotic regulators is being developed as possible therapies for cancer and other diseases 16,17. The development of model transgenic animals overexpressing pro-survival peptides supports such strategies 18.

Following the initial identification of a PCD pathway in the worm *C. elegans*, it became clear that PCD was conserved in multicellular organisms 6,19. The identification of conserved pathways in single celled organisms, including numerous species of yeast, has led to novel insights into the diversity of PCD pathways 20. More elaborate insights into the basic PCD processes have been uncovered in extensive studies that have now been carried out using the genetically tractable yeast *Saccharomyces cerevisiae* 18,21,22,23. In effect, yeast has proven useful for the study of all sub-forms of PCD 21,24,25,26,27. Yeast undergo PCD in response to a variety of different stresses and they display the typical hallmarks of cell death that can be easily monitored 25. Yeast also have apparent functional orthologues to a number of mammalian cell death regulators including caspase (*MCA1*), *AIF*, EndoG (*Nuc1*) and *YBH3,* a single BH3 domain Bcl-2 protein 28,29,30,31,32. So it is not surprising that yeast has proven to be an effective model for PCD processes including many neurological diseases such as Huntington’s and Parkinson’s disease 23. Thus the expression of human α-synuclein leads to cell death in yeast in a manner that is analogous to what is seen in Parkinson’s 33. More recent studies have suggested that increased calcium, mitophagy and specific genes including EndoG may be involved in mediating the toxicity of α-synuclein 30,34,35. Further humanized yeast is proving to be an excellent model to screen for drugs that can prevent cell death in response to PCD inducing conditions, including the heterologous expression of specific human genes 36,37,38. Similarly, yeast has proven effective in examining the effects of specific cancer drugs or to screen for drugs that may be therapeutically useful as chemotherapeutics 39.

We and others have used yeast conditionally expressing mammalian pro-apoptotic Bax as a model system to study PCD and as a platform to screen for and identify novel negative regulators 40,41,42,43,44,45,46. Many of the pro-survival genes identified in these heterologous screens have been shown to be pro-survival in their native mammalian hosts 40,47. Here we have identified a cDNA encoding human Thyroid Cancer-1 (hTC-1) 48 as a peptide capable of preventing the inhibitory effects of Bax in yeast. We further show that this peptide retains its pro-survival functions in yeast in response to other stresses including copper and activated pro-apoptotic peptides. These results are consistent with the observation that TC-1 is up-regulated by and serves to inhibit stress and apoptosis 49,50,51. Thus, yeast is a useful model to characterize potential cell survival functions of genes that are up-regulated in cancer.

## RESULTS AND DISCUSSION

**Figure 1:**
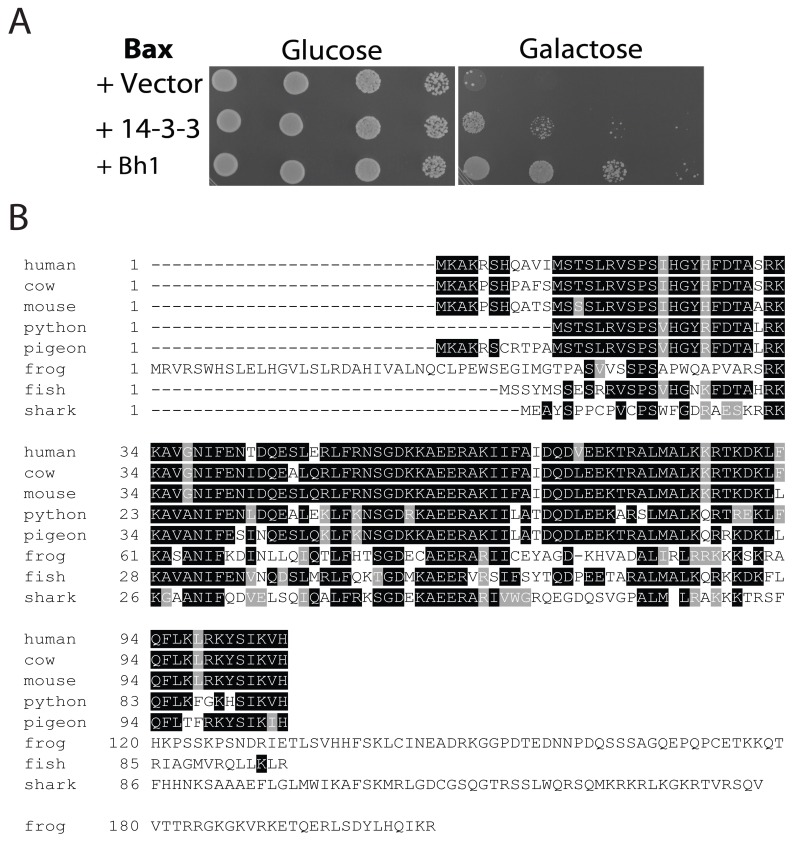
FIGURE 1: Identification of human TC-1 as a Bax suppressor that protects against programmed cell death-inducing levels of copper. **(A)** Plasmid DNA with the Bh1 sequence was obtained from the clone identified from the original cDNA library screen for Bax suppressors 52. It was then reintroduced into naïve BY4742 yeast cells with and without Bax. A human 14-3-3β/α cDNA was used as a positive control 53. Cultures of the transformants were grown, serially diluted, and spotted onto YNB media with glucose or with galactose. The plates were allowed to grow at 30°C and photographs of the plates are shown. **(B)** The amino acid sequence of human TC-1 was used to search the available protein databases at NCBI. Boxshade of the TCoffee alignment of representative sequences from different species showing strong similarity with hTC-1 are shown. The black shading indicates identical residues while grey indicates conserved residues. The scientific names and database accession numbers of the species used are as follows: human: *Homo sapiens* (Accession Number NM_020130); cow: *Bos taurus*(Accession Number NP_001030567); mouse: *Mus musculus* (Accession Number NP_081207); python: *Python bivittatus* (Accession Number XP_007430414.1); pigeon: *Columba livia* (Accession Number XP_005510897.1); frog: *Xenopus tropicalis* (Accession Number XP_002936483.2); fish: *Poecilia formosa* (Accession Number XP_007573497.1); and shark: *Callorhinchus milii* (Accession Number XP_007883063.1).

In a previous screen of a human cardiac cDNA library, Bh-1 was identified as a Bax suppressor 52. The Bh-1-expressing plasmid was isolated from the original yeast transformant and retransformed, along with the Bax expressing plasmid into the wild type strain. Cultures of cells harbouring Bax and the empty vector, Bax and 14-3-3β/α or Bax with Bh-1, were serially diluted and spotted onto nutrient agar media with glucose or galactose (Figure 1A). The human 14-3-3β/α cDNA was used as a control as it had been previously identified as an anti-apoptotic peptide 53. In Figure 1A, all three transformants show the same growth on glucose media. In contrast, cells harbouring Bax and the vector alone showed reduced growth when spotted onto the galactose-inducing media, as the pro-apoptotic Bax-expressing plasmid was activated. The co-expression of the Bh1-peptide along with the Bax (Bax+Bh-1) showed enhanced cell growth on the galactose media, indicating that Bh-1 is capable of suppressing the effects of Bax. The 0.8 kb Bh1 sequence contained an entire open reading frame from nucleotide 34 to 351 that was predicted to encode a 106 residue protein. The peptide’s sequence was compared to the proteins in the GenBank human database at NCBI. The sequence is identical to the 106 residue protein that is encoded for by a 1,841 bp cDNA (with a coding sequence between nt 66-386), corresponding to the human Thyroid Cancer-1 (TC-1) gene, also known as chromosome 8 open reading frame 4 (C8orf4) (Accession Number: NM_020130). Thus, the Bh-1 sequence corresponds to human TC-1.

In order to identify possible orthologues of the human TC-1 gene, the sequence was compared to the protein sequences available in the GenBank database. TC-1 peptides were found in multiple mammalian species as well as lower vertebrates including birds, reptiles, amphibians, sharks and fish. A representative sequence from each group was selected and these were then aligned with TCoffee and reformatted with BoxShade (http://www.ch.embnet.org/software/BOX_form.html) 54 (Figure 1B). The similarity between human TC-1 is nearly identical over the entire protein to other mammalian species 55. The similarity subsequently decreases further away from human, with the most divergent species from human, but still containing the TC-1 domain, being the elephant shark (37% identity over 80 residues). Earlier chordates, such as lampreys and hagfishes, were also searched for sequences having similarity to TC-1. However, the search returned sequences that had very little similarity. The last similar species, the shark, belongs to the jawed-vertebrates (gnathostomes- a sub-phylum of the vertebrata), which are known for having jaws, several types of mineralized bone, an adaptive immune system and limb development. This indicates that the introduction of the TC-1 gene appears to be between the divergence of the agnatha and the gnathostomata of the vertebrata phylum (550-450 MYA) 56.

In order to determine the most similar yeast proteins to TC-1, the yeast and fungal databases from www.yeastgenome.org were examined. It revealed three proteins encoded by *YGR238C*, *YGR198W* and *YIR001C*, which are suspected to negatively regulate mitotic exit, affect cargo transportation in endocytosis and bind to RNA in the cytoplasm, respectively. From these, the strongest case for a TC-1-like sequence is with *YIR001Cp*, an RNA-binding protein that modulates cytoplasmic mRNA. This protein is of comparable size, as it is 245 amino acids, while the other two are 882 and 817 amino acids, respectively. The sequence of *YIR001Cp *has limited similarity from residues 1 to 90 with the human protein (11 out of 90 identical and 16 out of 90 with conserved amino acids). It therefore seems unlikely that the yeast protein, *YIR001C*p is a TC-1 protein. It remains possible that a functional TC-1 orthologue exists in yeast, but this sequence diverged so much from its ancestral TC-1 that it does not have very direct sequence conservation with TC-1. Discerning subtle similarities between evolutionary distant ancestors has been successful in identifying a number of yeast orthologues to key mammalian apoptotic regulators, including *YBH3* and *YCA1* 28,29.

Given that alternative splicing is a common occurrence in genes encoding apoptotic regulators 57,58, we also examined the human TC-1 gene structure, with regards to its intron and exon organization. The TC-1 mRNA sequence was compared to the entire human genome, using the available database at NCBI. The analysis returned one continuous piece of DNA containing the entire TC-1 cDNA located on chromosome eight (Accession Number: AC_000140.1, between position 38544322 to 38546161 bp). The gene and cDNA sequences were continuous with each other, indicating that the entire cDNA was encoded for by a single exon. Therefore, TC-1 does not appear to have any splice-variants.

**Figure 2:**
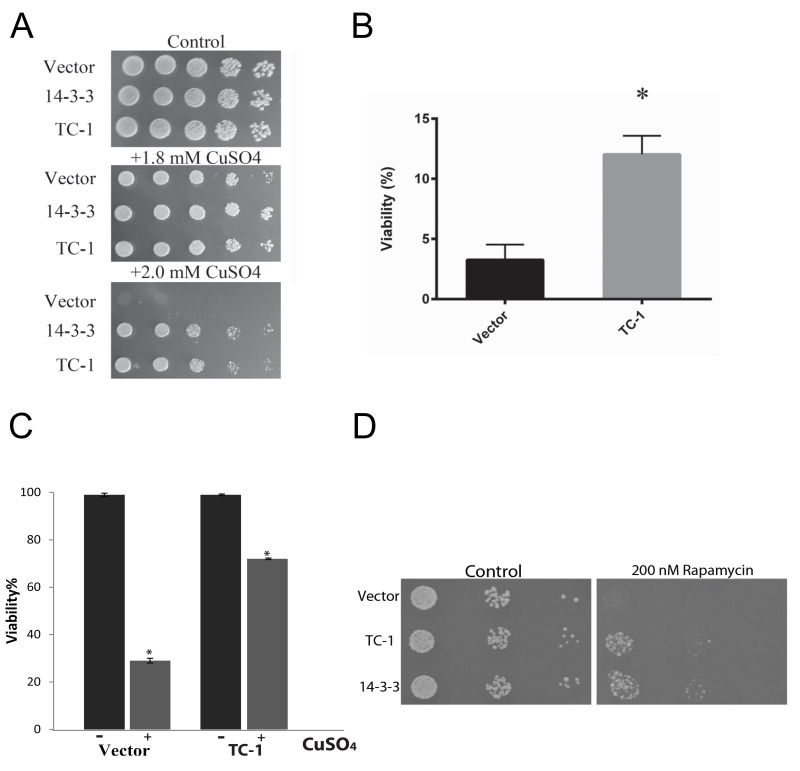
FIGURE 2: TC-1 protects against copper. **(A)** The growth of yeast cells transformed with empty plasmid (Vector), the TC-1 or h14-3-3 expressing plasmids was examined using a spot assay on YNB media with galactose alone or with the indicated concentrations of copper sulfate. The cells were allowed to grow at 30°C for 3 days and photographs of the plates are shown. **(B, C)** Survival of yeast cells transformed with empty plasmid (Vector) or the TC-1 expressing plasmid was measured after treatment with copper sulfate using a (B) clonogenicity assay or (C) by microscopical examination of cells stained with the vital dye trypan blue. Cells were grown in galactose-inducing liquid media with 1.4 mM copper sulfate for 18-20 h. For the clonogenicity assay, aliquots of cells were plated onto nutrient agar and allowed to grow for 48 h at 30°C. The number of colonies formed were counted and compared to the corresponding control. Alternatively, aliquots of cells were treated with trypan blue and viability was determined by microscopical examination of at least 300 cells. Data represents median ± SEM of at least n = 3 independent experiments. * indicates that the viability of cells with the TC-1-expressing plasmids was significantly higher than the viability of control cells (Vector); a P-value of 0.0128. **(D)** A spot assay was carried out using serially diluted cells obtained from freshly growing cells transformed with empty vector, hTC-1 or 14-3-3 expressing plasmids. Aliquots of the cells were plated onto nutrient agar media without (Control) or with 200 nM rapamycin.

In order to determine whether TC-1 could protect against other stressors besides Bax, cell-death-inducing levels of copper sulfate were used 59. Using the spot assay with increasing concentrations of copper sulfate, the cells harbouring only the vector show reduced growth, while cells harbouring TC-1 and 14-3-3 expressing plasmids remain largely unaffected (Figure 2A). In order to assess the ability of cells to survive exposure to stress and remain viable, cells were exposed to copper sulfate in liquid media for 20 h and their viability was determined using a clonogenicity assay. The viability of the empty vector control cells was reduced to 3.3 ± 2.2%, whereas the viability of cells expressing TC-1 was significantly higher at 12 ± 2.7%, an approximate 4-fold increase in viability in comparison to the vector (Figure 2B). We also carried the same experiment and directly evaluated viability after copper treatment by microscopical examination of cells stained with the vital dye trypan blue. In this assay, the viability of control cells decreased to 29.3 ± 2.1% with copper while the viability of TC-1 expressing cells was not as affected by copper as the viability was found to be 71.8 ± 1.9% (Figure 2C).

Although both methodologies yield similar results, there are notable differences in the apparent viabilities using clonogenicity and vital dye assays. This reflects differences in the timing and of the end points monitored as discussed in 57,58. Finally we evaluated the general nature of the pro-survival function of TC-1 by examining its ability to prevent the effects of rapamycin. Rapamycin is a well known inducer of autophagy that also induces PCD, that resembles apoptosis 53. Using the spot assay we show that control cells show reduced ability to grow on nutrient agar media that contains rapamycin (Figure 2D). As previously described, human 14-3-3 can confer partial resistance to rapamycin 53. Cells expressing TC-1 also have increased resistance to rapamycin indicating that TC-1 can protect cells from a variety of different stresses. Taken together these results indicate that TC-1 is capable of preventing stress-mediated cell death. Further the pro-survival properties of hTC-1 in yeast are consistent with the known stress-reducing and anti-apoptotic properties of this protein in mammalian cells 49,50,51. Thus yeast is a unique platform that may be useful in delineating the mechanisms by which TC-1 protects cells from stress. This is of potential clinical value since hTC-1 is reported to be transformative and may be a key tumor promoter in certain cancers 51,60,61,62. Thus yeast expressing hTC-1 may also serve as a platform to screen for novel inhibitors that may have therapeutic value 39.

**Figure 3:**
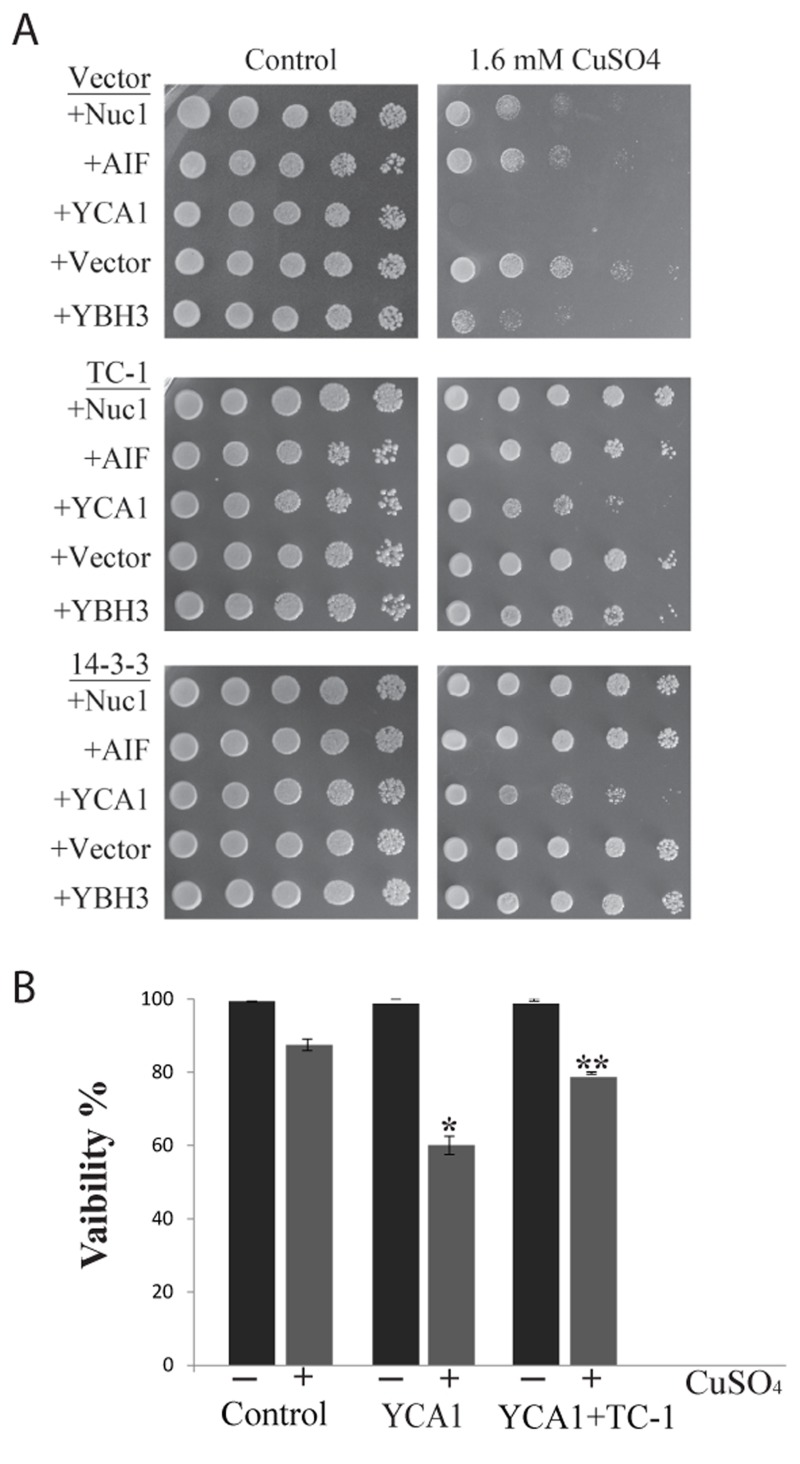
FIGURE 3: TC-1 protects against pro-apoptotic genes. **(A)** Yeast cells harbouring control plasmid (Vector) or plasmids expressing different pro-apoptotic peptides including *NUC1*, *AIF*, *YCA1* or *YBH3* were co-transformed with either empty plasmid (Vector) or a plasmid expressing TC-1 or 14-3-3. The spot growth assay was used to assess the growth of the transformants on YNB media with galactose alone (Control) or with a sublethal concentration of copper sulfate (CuSO_4_). The cells were allowed to grow at 30°C for 3 days and photographs of the plates are shown. **(B)** Freshly growing cultures of yeast transformants with two empty vectors (Control), one empty vector and a *YCA1* expressing plasmid (YCA1) or the *YCA1* and the TC-1 expressing plasmids (YCA1+TC-1) were challenged without (-) or with (+) sublethal *Yca1*p activating levels of copper (CuSO_4_). Viability was determined by microscopical examination of at least 300 cells. The data is shown as the percentage of cells that survive and represents the median ± SEM of at least n = 3 independent experiments. * indicates that the viability of cells expressing *YCA1* alone was significantly lower than the viability of control cells while ** indicates that the viability of cells expressing YCA1+TC-1 was significantly higher than the viability of cells expressing *YCA1* alone with P-values < 0.05.

There is a number of common processes that get activated by stress in yeast and mammalian cells that occur in response to the activation of specific pro-apoptotic proteins 21,25,63. To determine possible specific functions of TC-1, we generated yeast transformants that overexpress key pro-apoptotic peptides including* NUC1*, *AIF*, *YCA1* and *YBH3* with and without TC-1. *NUC1* encodes Nuclease 1 (EndoG), a protein known to translocate from the mitochondria to the nucleus in order to degrade DNA under stressful conditions 64, while *AIFp *is Apoptosis-Inducing Factor, a protein known to be released from the mitochondria under stressful conditions and mediates cell death by degrading DNA 31. *YCA1p* (also called *MCA1p*) is Yeast Caspase-1, a metacaspase that regulates apoptosis upon induction of a stress such as hydrogen peroxide and acetic acid 29. *YBH3p* is Yeast Bax Homology 3 protein that is a Bcl-2 family protein and induces PCD 28. Sub-lethal level of a mild stress is often required to activate over-expressed native pro-apoptotic proteins for them to induce cell death. Here we used copper sulfate at a concentration that is known to be sub-lethal 59. Cultures of yeast doubly transformed with different combinations of expression vectors, including empty vectors, pro-apoptotic genes and TC-1 or 14-3-3 were serially diluted and spotted on nutrient agar media containing galactose alone or with sub-lethal copper sulfate (Figure 3A). The inhibitory effects observed with all four pro-apoptotic genes, was compensated for by the over-expression of TC-1 or 14-3-3 (Figure 3A). Thus, hTC-1 was able to protect against the effects of multiple pro-apoptotic genes. We confirmed the results of the spot assays using vital dye based assays. Cultures of yeast cells having both empty vectors, empty vector and *YCA1* overexpressing plasmid as well as yeast having *YCA1* and TC-1 expressing plasmids were challenged with low level of copper in order to activate the overexpressed *Yca1p* and viability was determined 18 hours later. Yeast expressing *YCA1* had a lower survival rate (62.0 ± 6.2%), compared to control cells with both empty vectors (87.4 ± 2.8%) (Figure 3B). This reduced viability, induced by activated *Yca1p* was mitigated by the co-expression of TC-1 as cells expressing both TC-1 and *YCA1* had increased survival rates (78.8 ±0.5%), compared to yeast expressing *YCA1* alone (62.0 ±6.2%) (Figure 3B).

**Figure 4:**
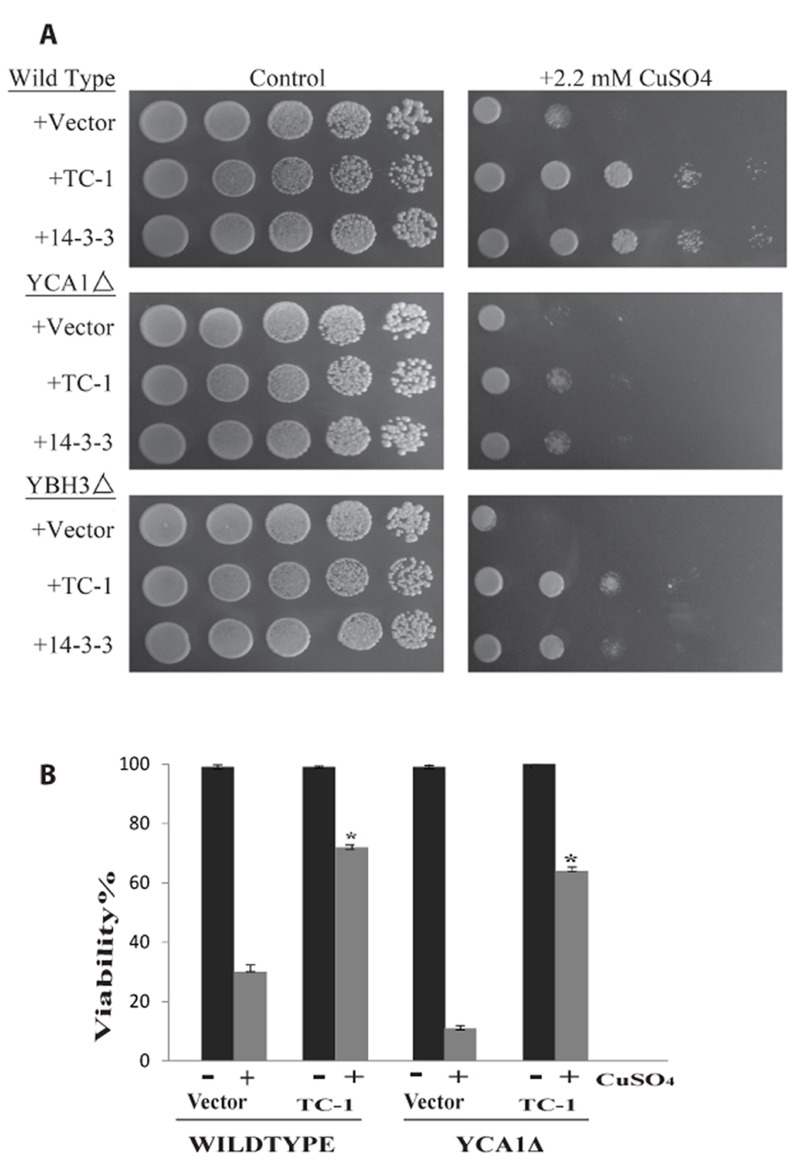
FIGURE 4: TC-1 protects in the absence of pro-apoptotic genes. **(A)** The growth of wild type yeast as well as mutants lacking *YCA1* or *YBH3* transformed with empty plasmid (Vector) or the TC-1 or 14-3-3 expressing plasmids was examined using a spot assay on YNB media with galactose alone or with the indicated concentrations of copper sulfate. The plates were allowed to grow at 30°C for 3 days and photographs of the plates are shown. **(B)** Galactose growing cultures of wild type yeast as well as mutants lacking *YCA1* transformed with empty plasmid (Vector) or the TC-1 expressing plasmid were grown for 18 hours without (-) or with (+) 1.4 mM copper sulfate (CuSO_4_). Aliquots of the cells were stained with trypan blue and viability was determined by microscopical examination. Viability was determined by microscopical examination of at least 300 cells. The data is shown as the percentage of cells that survive and represents the median ± SEM of at least n = 3 independent experiments. * indicates that the viability of cells TC-1 was significantly higher than the viability of control cells (P-values < 0.05).

In our hands the overexpressed *YCA1 *and *YBH3* were the most lethal and their effects were reversed by TC-1 (Figure 3A). In order to examine the possibility that TC-1 may be inhibiting a specific *YCA1p *and/or *YBH3p *pathway, we investigated if the knockout of *YBH3* or *YCA1* would affect the ability of TC-1 to protect the cell. Cultures of wild type as well as *YCA1*Δ and *YBH3*Δ mutants harbouring the vector, TC-1, or 14-3-3-expressing plasmids, were spotted onto nutrient agar plates with and without copper stress (Figure 4A). TC-1 was able to overcome the inhibitory effects of copper in all three strains.

We have previously reported that cells lacking *YCA1* are more sensitive to copper than wild type cells 65. This increased sensitivity to copper, seen in Figure 4A, serves to decrease the apparent protective effect of TC-1. We thus used vital dye to further examine the effect of TC-1 on copper mediated cell death in *YCA1*Δ cells. The viability of *YCA1*Δ cells treated with copper is decreased to 11.1 ± 1.7% while the viability of these mutant cells is increased to 63.7 ± 2.9% by the expression of TC-1 (Figure 4B). Taken together these results indicate that TC-1 is not specific to *YCA1p* or *YBH3*p pathways and may be indicative that TC-1 is acting downstream of many different pro-apoptotic proteins.

Other studies have also seen the protective effects of TC-1. It was demonstrated that TC-1 binds to the β-cantenin binding nuclear protein Chibby. Given that Chibby is a negative regulator of β-cantenin, TC-1 thus enhances Wnt pathway and increases cell proliferation 66. However, yeast is not known to have a Wnt-signaling pathway, indicating that the human TC-1 gene is capable of activating an additional pathway that is present in yeast 67. Another possibility is that TC-1 may act as a regulator of the heat shock response 49. This may indicate why TC-1 is capable of protecting against a myriad of pro-apoptotic stresses and proteins, and why some of the effects observed of TC-1 are similar to the effects of 14-3-3, a chaperone type protein 53.

## MATERIALS AND METHODS

### Yeast Strains, Plasmids and Growth

The *Saccharomyces cerevisiae *BY4742 (MATα his3∆1 leu2∆0 lys15∆0 ura3∆0) strain was used for the wild type strain. The *YCA1*∆ and *YBH3*∆ deletion strains were isogenic to BY4742 59. The plasmids expressing the cDNAs for human 14-3-3β/α and TC-1 (Bh1) as well as yeast *YBH3*, *YCA1*, *AIF1* and *YBH3* expressed under the control of the *GAL1*-galactose-inducible promoter, were previously isolated and described 28,29,30,31,52,53. The plasmids were introduced into yeast using the lithium acetate method and were selected for, and maintained by, the omission of the appropriate nutrient on YNBD (1% yeast nitrogen base (YNB), 2% glucose and the required amino acids). In order to express the plasmid under the influence of the *GAL1* promoter, yeast were grown on YNBGal/Raf (1% YNB, 1% raffinose and 2% galactose).

### Spot and Viability Assays

Yeast transformants that were maintained on YNBD media with supplements, were used to inoculate liquid YNBD and were incubated at 30°C for 18-24 h with shaking. The freshly saturated cultures were then used to inoculate liquid YNBGal/Raf (1 in 20) and incubated at 30°C for 4-6 h with shaking. The cultures were then serially diluted (1:5) with sterile distilled water and 10 μL of each dilution was spotted onto agar plates, and subsequently incubated at 30°C for three days. Spot assays were repeated for a minimum of three times. Any substance, such as copper sulfate, was sterilized before its addition to the agar plates. For viability assays, freshly saturated cultures were then used to inoculate liquid YNBGal/Raf (1 in 100) and incubated at 30°C with shaking with 1.4 or 1.6 mM copper sulfate (CuSO_4_) was added to the cultures and then incubated at 30°C for 18-20 h with shaking. The cultures were then serially diluted, spread onto nutrient agar plates, incubated at 30°C for 48 h and the number of colonies determined 58. Viability assays were repeated for a minimum of three times and a minimum one hundred colonies were counted for each assay. Alternatively the cells were stained with the vital dye for 10 minutes then viability was determined by microscopical examination of at least 300 cells in three different experiments 59. In order to activate the proteins that are overexpressed from the different pro-apoptotic genes, the cells were treated with sub-lethal levels of copper (1.6 mM for agar plates and 1.0 mM for liquid cultures).

### Statistical Analysis 

The two-tail one-way ANOVA was performed using GraphPad by Prism in order to assess for statistical significance.
